# Natural Immunomodulators

**DOI:** 10.1155/2017/7529408

**Published:** 2017-12-14

**Authors:** D. Ortuño-Sahagún, K. Zänker, A. K. S. Rawat, S. V. Kaveri, P. Hegde

**Affiliations:** ^1^Instituto de Investigación en Ciencias Biomédicas (IICB), CUCS, Universidad de Guadalajara, Guadalajara, JAL, Mexico; ^2^Witten/Herdecke University, Witten, Germany; ^3^National Botanical Research Institute, Lucknow, Uttar Pradesh, India; ^4^Institut National de la Santé et de la Recherche Médicale (INSERM), Paris, France

In this special collection of research results, we present a group of original works performed by employing natural compounds as tools to approach immunomodulation at different levels and against different diseases.

Natural compounds have contributed enormously to immunomodulatory therapeutics. Since ancient times, natural medicines have constituted treatments with minimal side effects. There are thousands of natural compounds that are known to influence the immune system by either affecting the functions of immune cells or affecting antibody secretion to control the infection and to maintain immune homeostasis. The relevance of this research would be crucial to the search for better treatments, both to complement those that already exist and to develop new strategies for the prevention and treatment of immune-related diseases. Therefore, it is interesting to dissect the molecular mechanisms of the immunomodulatory effects of natural compounds and to discover novel promising candidates that can be used in the future immunotherapeutic strategies.

Immunomodulation is a key issue in tissue homeostasis for the physiological stability of organisms. Consequently, it is important to search for immunoregulators, such as those derived from natural immunomodulators, with less severe side effects. This is the case for the work of Y.-H. Cheng et al. in which the authors demonstrate Th-1 selective immunomodulatory activity for crude leaf extracts from *Neolitsea* spp., which contains phytochemicals meriting further research as potentials for development as selective immunomodulators. Additionally, M. O. Arruda et al. reported the modulatory activity of *Mentha piperita* (peppermint) leaf hydroalcoholic extract on macrophages, which are essential cells against bacterial infection, by attenuating their oxidative stress and improving their survival. Furthermore, S. Lewicki et al. explored the immunomodulatory properties of the *Rhodiola kirilowii* aqueous extract to stimulate innate immunity in an attempt to avoid, or to limit, the excessive use of antibiotics during pregnancy and lactation, which has been associated with a risk for immune system developmental disorders.

The immune system encounters one of its great challenges against cancer. In the beginning, cell proliferation dysregulations begin in a small microenvironment, and distinct immune cell populations are recruited to the vicinity of tumor [1], thus the high relevance of cancer immunotherapy and the need to conduct exploration for new natural compounds, or derived compounds, with antitumor properties, as that which the aqueous extract of *Viscum album* exhibits in the work presented by R. M. Stammer et al. against alveolar rhabdomyosarcoma. In addition, J.-T. Yang et al. demonstrated that propyl gallate, a phytochemical polyphenolic compound, can affect migration of malignant glioma cells through inhibition of ROS and the NF-*κ*B pathway.

Cytokines and chemokines regulate immune responses by signaling, through membrane receptors, whose signaling pathways can be evaded or mimicked by viruses [2]. Consequently, research on natural compounds that can prevent, reduce, or counter viral infective process acquires relevance, in diseases such as that presented by S. Feustel et al. in which an extract of *Moringa oleifera* exhibits protective effects against hepatitis B virus (HBV) infection, particularly for genotypes C and H.

The largest organ in the human body is the skin, and it represents the greatest upon which the immune system needs to act. Therefore, an imbalance or lack of adjustment of innate or adaptative immunity leads to immune diseases. Three of the works presented herein approach immunity modulation in this organ's epithelium, dealing with atopic dermatitis and psoriasis.

Atopic dermatitis is not a life-threatening disease; however, it severely affects patients' quality of life. Again, it is derived from immune-response deregulation and with an undefined etiology to date. Recently, air pollutants have been involved as inductive factors [3]; therefore, it became relevant to find some natural elements that can counteract this pathology, as F. C. Muñoz et al. present in their results on glycomacropeptide, a dairy bioactive peptide derived from milk, *κ*-casein, which possesses prebiotic, anti-inflammatory, and immunoregulatory properties that can lead to prophylactic and therapeutic actions.

Likewise, psoriasis is the most common type of chronic inflammatory dermatosis, derived from a disorder of the immune system. Several genetic-susceptibility factors have been discovered to be involved in this disease [4]. However, their treatment comprises a search for compounds able to prevent or thwart its effects. Here, C.-Y. Lai et al. present a comprehensive review focusing on the natural modulators able to inhibit endosomal TLR activation that can represent candidate drugs for developing novel treatment options for psoriasis. In addition, S. J. Kim et al. revealed promissory experimental results with the use of the *Euphorbia kansui* methanol extract, which ameliorates the symptoms of psoriasis through inhibition of Th17 differentiation and dendritic cell activation.

Undoubtedly, there remains much more work to be done, but this avenue of natural immunomodulators is one highly promissory pathway to continue to explore and investigate. Many more surprises are awaiting to be discovered in the prevention and treatment of inflammatory and immune-regulated diseases. In the future, an extensive investigation is required with respect to their mechanisms of action at the systemic, cellular, and molecular levels and extension to a broad spectrum clinical trial. Given the multiple biological effects of natural compounds, it is exceedingly interesting to design future therapeutic strategies for inflammatory pathologies and malignant diseases with a synergistic combination of natural products together and various conventional therapies. We hope that researchers enjoy the analysis of this special issue.
